# Supplementary material for: Mitochondrial membrane proteins and VPS35 orchestrate selective removal of mtDNA
Source: Nat Commun. 2022 Nov 7;13:6704. doi: 10.1038/s41467-022-34205-9 (PMC9640553; doi:10.1038/s41467-022-34205-9)
Supplement: Supplementary file 1 — Supplementary Information [file 41467_2022_34205_MOESM1_ESM.pdf]

Supplementary Information

Mitochondrial membrane proteins and VPS35 orchestrate selective removal of mtDNA.

Ayesha Sen, Sebastian Kallabis, Felix Gaedke, Christian Jüngst, Julia Boix, Julian Nüchel, Kanjanamas Maliphol, Julia Hofmann, Astrid C Schauss, Marcus Krüger, Rudolf J. Wiesner and David Pla-Martín

Supplementary methods

Molecular biology and vector generation

Twnk ORF was amplified from plasmids pJet2-Twinkle and pROSA-K320E[24] and cloned in pmCherry-N1. For retroviral vector generation, Twinkle and Twinkle-mCherry were amplified and subcloned into pLenti-CMV Puro DEST (Addgene #17452). All ORFs were subcloned into pBabe-Puro vector, kindly provided by Dr. Bernhard Schermer. pLX304-TWINKLE-APEX2 vector was kindly provided by Dr. Alice Ting. To generate *Mus musculus Twnk* vectors, *Homo sapiens TWNK* was replaced by *Twnk* ORF and subcloned into pBABE-Puro vector. Mitochondrial matrix APEX2 control vector (Addgene #72480) was also subcloned into pBABE-Puro. For generation of Tfam-GFP, SAMM50-HA and ATAD3-HA total RNA from mouse liver was isolated and converted into cDNA using TRIzol (Thermo Fisher) and RevertAid First Strand cDNA Synthesis Kit (Thermo Fisher) using PolyT as feeder. *Tfam* cDNA was cloned into pEGFP-N1; SAMM50 cDNA and ATAD3 cDNA were cloned in pJET1.2 using CloneJET PCR Clonning kit (Thermo Fisher). To generate SAMM50-HA, ΔPOTRA-SAMM50 (Δaa1-aa125), ATAD3-HA and ΔIMS-ATAD3-HA (Δaa1-aa109), ORFs were amplified from pJET1.2 with oligos containing HA sequence and subcloned in pBabe-Puro vector (Supplementary Table 1). VPS35-GFP was generated by PCR amplification from pLenti6/V5/DEST-VPS35 (Addgene #21691) and cloned into pEGFP-C1. *Atad3* KD and *Samm50* KD clones were generated by transducing MEFs with the vector pMKO1-GFP (Addgene #10676) containing a shRNA (Supplementary Table 1). Scramble shRNA was used as a control. For CRISPR Cas9 KO generation, two gRNA directed to exon 4 and exon 5 (Supplementary Table 1) were cloned in pSpCas9 (BB)-2A-Puro V2.0 (Addgene #62988). All generated vectors containing ORF were validated by Sanger sequencing.

Liquid chromatography – mass spectrometric analysis

Both affinity-enriched and pulsed SILAC proteomics samples were analysed in positive mode using data-dependent acquisition (DDA) either by an Easy-nLC 1000 – Q Exactive Plus or an Easy-nLC 1200 – Orbitrap Eclipse tribrid system (all Thermo Fisher). On-line chromatography was directly coupled to the mass spectrometric systems using a nanoelectrospray ionization source. Peptides were separated by reversed-phase chromatography with a binary buffer system of buffer A (0.1% formic acid in water) and buffer B (0.1% formic acid in 80% acetonitrile) using a 60 min chromatographic gradient for IP samples and 120 min for pulsed SILAC samples. Separation was performed on a 50 cm long in-house packed analytical column filled with 1.9 µM C18-AQ Reprosil Pur beads (Dr. Maisch). Using the 60 min chromatographic gradient peptide separation based on their hydrophobicity was performed by linearly increasing the amount of

buffer B from initial 13% to 48% over 35 min followed by an increase of B to 95% for 10 min. The column was washed for 5 min and initial column conditions were achieved by equilibrating the column for 10 min at 7% B. Full MS spectra (300 to 1750 m/z) were acquired with a resolution of 70,000, a maximum injection time of 20 ms and an AGC target of 3e6. The top 10 most abundant peptide ions were isolated (1.8 m/z isolation windows) for subsequent HCD fragmentation (NCE = 28) and MS/MS recorded at a resolution of 35,000, a maximum injection time of 120 ms and an AGC target of 5e5. Peptide ions selected for fragmentation were dynamically excluded for 20 seconds.

Using the 120 min chromatographic gradient peptides were separated by linearly increasing B from initial 4% to 25% over 96 min followed by an increase of B to 55% over 14 min. After a steep increase of B to 95% over 2 min the analytical column was washed for 8 min at 95% B. Full MS spectra (375 to 1500 m/z) were acquired with a resolution of 60,000, a dynamic injection time and an automated AGC target. The top 20 most abundant peptide ions (charge state 2 – 7) were isolated (1.2 m/z isolation windows) for subsequent HCD fragmentation (NCE = 30) and MS/MS recording at a resolution of 15,000, a maximum injection time of 22 ms and an automated AGC target. Peptide ions selected for fragmentation were dynamically excluded for 60 seconds.

Data processing and analysis for proteomics

All recorded RAW files were processed with the MaxQuant software suite [75] (1.5.3.8 for IP data, 1.6.14 for pSILAC). For peptide identification and scoring MS/MS spectra were matched against the mouse Uniprot database (downloaded 08/15/2019) using the Andromeda search algorithm [76]. For the affinity-enriched samples, multiplicity was set to one and trypsin/P was selected as digestive enzyme. Carbamidomethylation was set as a fixed modification and methionine oxidation or N-terminal acetylation was selected as variable modification. Peptides were identified with a minimum amino acid length of seven and a false-discovery rate (FDR) cut-off of 1% on the peptide level. Proteins were identified with FDR < 1% using unique and razor peptides for quantification. Label-free quantification was performed using the standard settings of the maxLFQ algorithm. Match between runs was activated. pSILAC data were analysed with the same settings with some modifications. Multiplicity was set to two with Lys6 as heavy isotope label. Lys-C/P was selected as digestive peptide and LFQ quantification was deactivated.

Statistical analysis and visualization were done with the Perseus (1.6.5) and InstantClue software suits [77,78]. LFQ intensities of IP samples were $\log_2$-transformed and filtered for proteins identified in at least two replicates of one condition. Missing values were imputed with the Perseus plugin ImputeLCMD using deterministic minimal value approach (MinDet, q-value = 0.001) to simulate the lower detection limits of the mass spectrometer. To evaluate principal components responsible for the variances between samples, we performed a principal component analysis. Further, we performed a two-sided Student's *t*-test to identify significantly regulated proteins (S_0 = 0.1, permutation-based FDR = 0.05, 500 randomizations). If not otherwise indicated, significantly enriched proteins were determined by a combination of FDR-corrected p-values and log2 protein fold changes (q-value < 0.05 and absolute log2 FC > 1). For enrichment

quantification, significant hits were filtered according to their annotation in MitoCarta, and grouped using their subcellular compartment annotated in GOCC. Z-score-normalized protein intensities were hierarchically clustered using Euclidean distances to visualize protein enrichments in different heat maps. We proceeded similarly for the pulsed SILAC data but used the heavy-to-light (H/L) ratios for statistical testing. We used the same filtering-criteria but did not impute missing values. We performed 1D annotation enrichments and Fisher exact tests (both p-value < 0.02, Benjamini-Hochberg FDR correction) to identify enriched categorical terms in the different conditions.

Electron microscopy

For APEX2 electron microscope cells were grown on small discs of aclar foil and fixed for 1 h in 2% Glutaraldehyde with 2 %Sucrose in HEPES buffer pH 7,4. After washing two times with 0.1M Cacodylate buffer, free aldehyde groups were quenched with 0.1M Glycine in 0.1M Cacodylate buffer twice for 20 min. After a short wash with 0.1M Cacodylate buffer, cells were incubated in 0.5mg/ml Diaminobenzidine in 0.1M Cacodylate buffer and, after 10 min, a final concentration of 0.03% H_2O_2 added and incubated for 30 min. Finally, cells were washed thrice with 0.1M Cacodylate buffer and incubated with 1% Osmiumtetroxid and 1.5% Potassium hexacyanoferrat for 30 min at 4°C. After 3x 5 min washes with ddH2O, samples were dehydrated using ascending ethanol series (50%, 70%, 90%, 100%) for 5 min each and infiltrated with a mixture of 50% Epon/ethanol overnight at 4°C and with pure Epon for 2 h twice. Samples were embedded into TAAB capsules and cured for 48 h at 60°C.

For correlative Light-Electron Microscopy (CLEM), cells transduced with Twinkle-mCherry were grown in glass bottom dishes (MatTek), coated with a carbon finder pattern and transfected with VPS35-GFP plasmids as described previously. Cells were fixed for 30 min at room temperature and 30 min at 4°C in 2% glutaraldehyde, 2.5 % sucrose, 100mM $CaCl_2$ in 0.1M HEPES pH 7.4 and washed with 0.1M HEPES buffer. Fluorescent images were taken using the SP8 confocal microscope (Leica) with 63x/1.40 oil objective and brightfield images were used to localize the coordinates of cells of interest. Following light imaging, cells were incubated with 1% Osmiumtetroxid for 30 min at 4°C, washed in 0.1M Cacodylate buffer and dehydrated using ascending ethanol series (50%, 70%, 90%, 100%) at 4°C. Cells were infiltrated with a mixture of 50% Epon/ethanol for 1 h, 66% Epon/ethanol for 2 h and pure Epon overnight at 4°C. TAAB capsules filled with Epon were placed upside down onto the glass bottom and cured for 48 h at 60°C. Glass bottom was removed by alternatingly putting the dish into boiling water and liquid nitrogen. Block face was trimmed to the previous noted coordinates using a razor blade and ultrathin sections of 70 nm were cut using an ultramicrotome (Leica Microsystems, UC6) and a diamond knife (Diatome, Biel, Switzerland), stained with 1.5 % uranyl acetate for 15 min at 37°C and lead citrate solution for 4 min.

For immunogold staining (Tokuyasu technique), cells were fixed using 2% formaldehyde and 0.2% glutaraldehyde in 0.1M PHEM buffer for 30 min at RT and 30 min at 4°C followed by wash in 0.1% Glycine/PBS. Cells were scraped in 1% gelatin/PBS, washed two times in 0.1%

glycine/PBS, resuspended in 10% gelatin/PBS, incubated at 37°C for 10 min and cooled down on ice for 30 min. Gelatin cubes were stored overnight in 2.3M sucrose at 4°C. Gelatin pieces were mounted on aluminum pins for cryo-ultramicrotomy and snap-frozen in liquid nitrogen. Ultrathin cryo-sections of 70 nm were cut with a diamond knife (Diatome, Biel, Switzerland) using a Leica UC6 with FC7 at -115°C. Sections were recovered in ice cold 1:2 mixture of 2% methylcellulose/2.3 M sucrose and transferred onto 100 mesh formvar coated copper grids. Gelatin was removed by incubating grids on PBS for 1h at 40°C following PBS wash. Free aldehyde groups were inactivated by a 15 min wash with 0.05M glycine/PBS. Grids were incubated on a drop of blocking solution for protein A-gold, washed with 0.1% BSA-c/PBS, and mouse monoclonal α-VPS35 (1:50) in 0.1% BSA-c/PBS for 90 min, followed by washes in 0.1% BSA-c/PBS. Protein A-gold (10 nm) was incubated for 90 min diluted 1:20 in 0.1% BSA-c/PBS, post fixed 5 min with 2% glutaraldehyde and contrasted for 5 min with 0.4% uranyl acetate in 2% methylcellulose on ice.

Supplementary Tables

Supplementary Table 1. shRNA oligonucleotides

**shRNA name**	**Sequence 5´-3´**
Atad3 shRNA	GCAGTTTGATTGGGCTATCTTCTCGAGGATAGCCCAATCAAACTGCTTTTTTG
Scramble shRNA	CCTAAGGTTAAGTCGCCCTCGCTCGAGCGAGGGCGACTTAACCTTAGGTTTTTG
Samm50 shRNA	GAGGAGATGTGAGCTTCATTTCAAGAGAATGAAGCTCACATCTCCTCTTTTTG

Supplementary Table 2. PCR oligonucleotides

	**Sequence 5´-3´ Forward**	**Sequence 5´-3´ Reverse**
m*Vps35*-ex4-104fw-s	CACCGTATGAACTTGTACAGTACGC	AAACGCGTACTGTACAAGTTCATAC
m*Vps35*-ex5-91fw-s	CACCGATTTGGTAGAAATGTGCCG	AAACCGGCACATTTCTACCAAATC
*Vps35*_Ex4	CTGAGCCAGGAGATCATGAATTC	GCTTCCACTACTGAGCTAGATCAC
*Vps35*_Ex5	GTCTAGACACAACTTACTGACACC	GTAGTGTGTTTGAATACAGTCAAG
Del983-4977 Forward	TCGTAACAAGGTAAGCATACTG	CTCGCGGACTAGTATATCCT
mtDNA copy number	CCTATCACCCTTGCCATCAT	GAGGCTGTTGCTTGTGTGAC
nucDNA-mtDNA copy number	ATGGAAAGCCTGCCATCATG	TCCTTGTTGTTCAGCATCAC
mitoLongRange	GTTCAACGATTAAAGTCCTACGT	GTTGTTTGATCCTGTTTCGTG
mtDNA Short LR-rtPC	CCCAGCTACTACCATCATTCAAGT	GATGGTTTGGGAGATTGGTTGATGT
mtDNA Long LR-rtPC	GCCAGCCTGACCCATAGCCATAATAT	GATGGTTTGGGAGATTGGTTGATGT

Pgc1α qPCR	AAGTGTGGAACTCTCTGGAACTG	GGGTTATCTTGGTTGGCTTTATG
Atad3 qPCR	GCCAGACCATCTTGGAGTCT	TACCGACCAGCAACAGAAGT
Samm50 qPCR	ATGCCATGGTCATCGACTCT	ACGTGGAGAATACCGAGTCC
Gapdh qPCR	AGGTCGGTGTGAACGGATTTG	TGTAGACCATGTAGTTGAGGTCA
Asc qPCR	GAAGCTGCTGACAGTGCAAC	GCCACAGCTCCAGA CTCTTC
Ifnb qPCR	CCCTATGGAGATGACGGAGA	CCCAGTGCTGGAGAAATTGT
Nlrp3 qPCR	CGAGACCTCTGGGAAAAAGCT	CATACCATAGAGGAATGTGATGTACA
Ifit1 qPCR	CTGAGATGTCACTTCACATGGAA	GTGCATCCCCAATGGGTTCT
Stat1 qPCR	CGCGCATGCAACTGGCATATAACT	ATGCTTCCGTTCCCACGTAGACTT
Usp18 qPCR	AGAGTTAGCAAGCTCCGACAT	TGAGGTGAATGGTCAAGGTTTG

Supplementary Figures

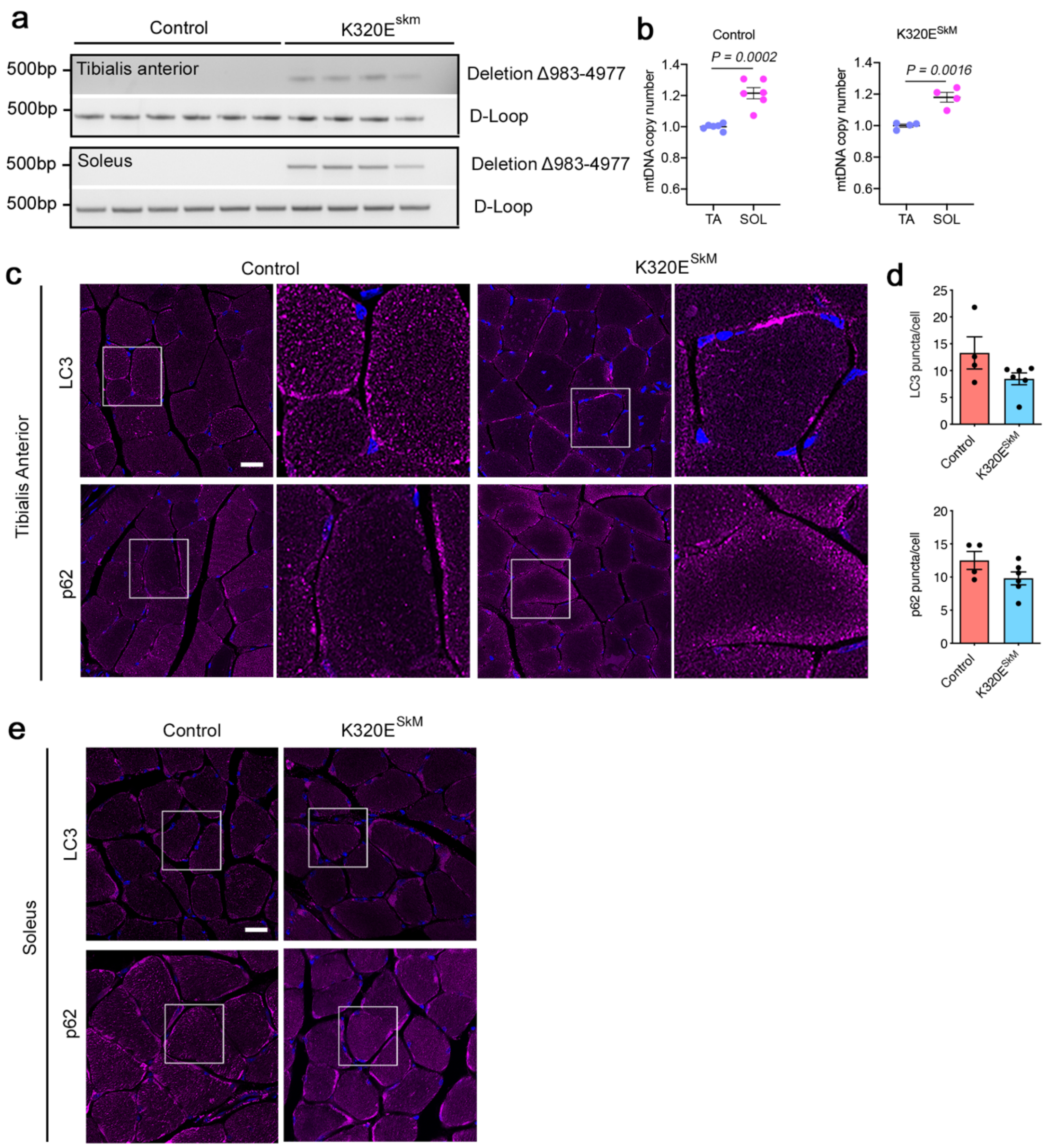

Supplementary Figure 1. (**a**) Conventional PCR with specific oligonucleotides flanking the deletion mtDNA-Δ983-4977 and (**b**) mtDNA copy number in muscles from 24 months old control and Twinkle-K320E mice. This graph shows a comparison between muscles different from the samples analysed in Fig. 1b. (**c-e**) *In situ* immunofluorescence and image quantification showing autophagic markers LC3 and p62 in cryosections of M. tibialis anterior and (**e**) of M. Soleus. Magnified areas for M. Soleus are shown in Figure 1f. 5 random pictures with 4 fibers per picture were analysed per animal to obtain averaged values. (Control: n=4; K320E: n=5). Scale bar, 20 µm. *P* values calculated using Unpaired Student´s T-test. Data is presented as Mean ± SEM.

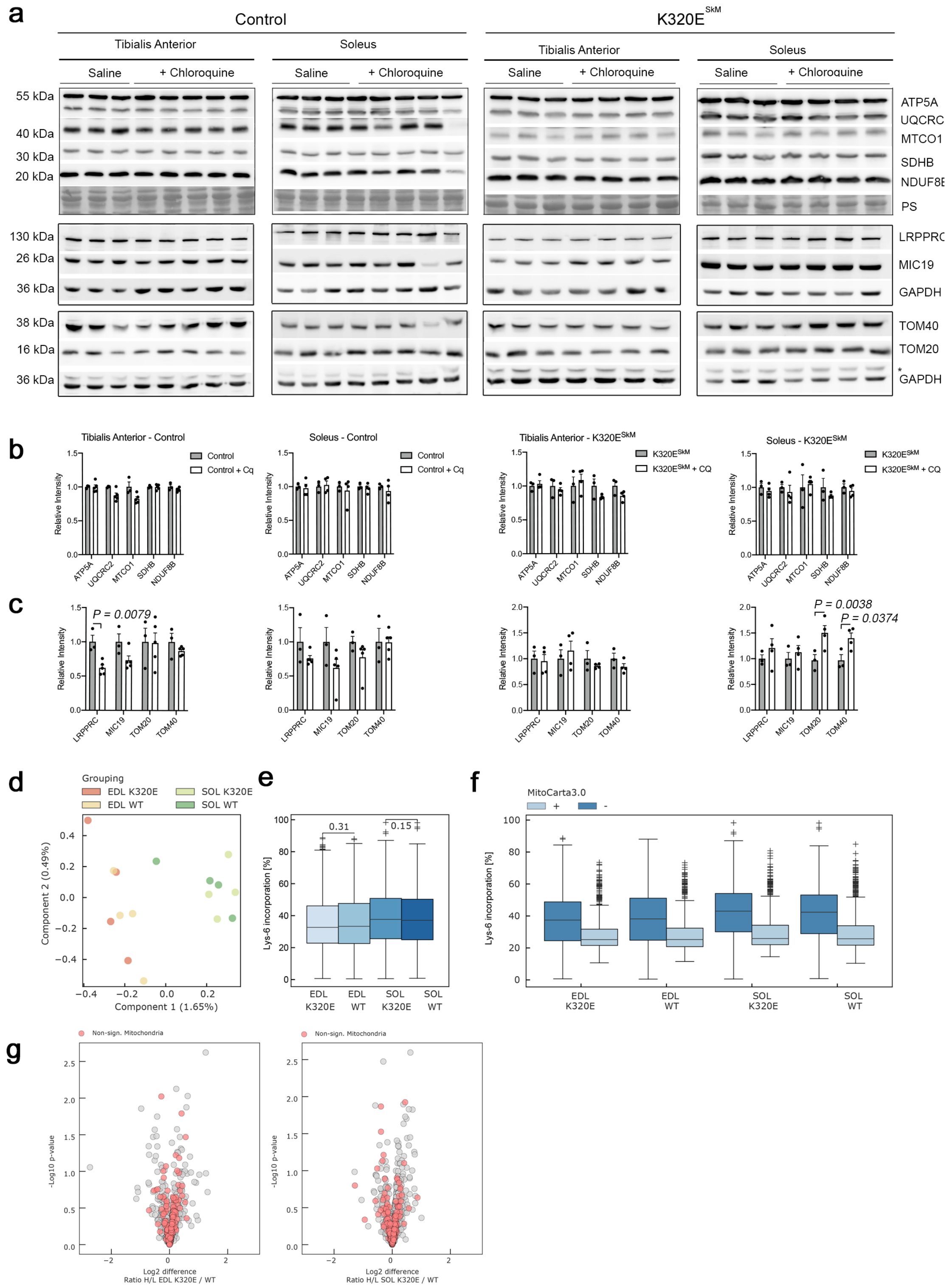

Supplementary Figure 2. (**a-c**) Western Blot analysis and relative quantification of mitochondrial proteins for SOL and TA in steady state and after 4h lysosomal block with chloroquine. GAPDH was used as a loading control except for OXPHOS related proteins, where Ponceau S (PS) was used. *, TOM40 signal. (n=3-5 mice per condition). *P* values calculated using Unpaired Student´s T-test. Data is presented as Mean ± SEM. (**d**) Principal component analysis of log2 H/L ratios of *in vivo* pulse SILAC-labelled proteins identified in M. extensor digitorum longus (EDL) or M. soleus (SOL) tissue. (**e**) Box plot for Lys-6 incorporation

rates in muscles from Twinkle-K302E and Twinkle-WT mice. (**f**) Box plot for MitoCarta annotated proteins (light blue) and all proteins (dark blue) labelled with Lys-6. Boxes in boxplots from (**e, f**) indicate the interquartile range (IQR) of data with the 25% quartile as minimal and the 75% quartile as the maximal limit. The median is indicated by a horizontal line. Whiskers incorporate all values within the upper/lower 1.5-fold IQR. All outliers are indicated by individual crosses. (**g**) Volcano plots comparing log2 H/L ratios of *in vivo* pulse SILAC-labelled mitochondrial and non-mitochondrial proteins in Twinkle-K320E M. extensor digitorum longus (EDL) and M. soleus (SOL) versus Twinkle-WT. Red dots: mitochondrial proteins; Significantly changed H/L ratios between Twinkle-WT and Twinkle-K320E were not detected (significance: q-value < 0.05 and abs. log2 fold change ratio H/L > 1). For proteomics analysis n=4 mice per genotype.

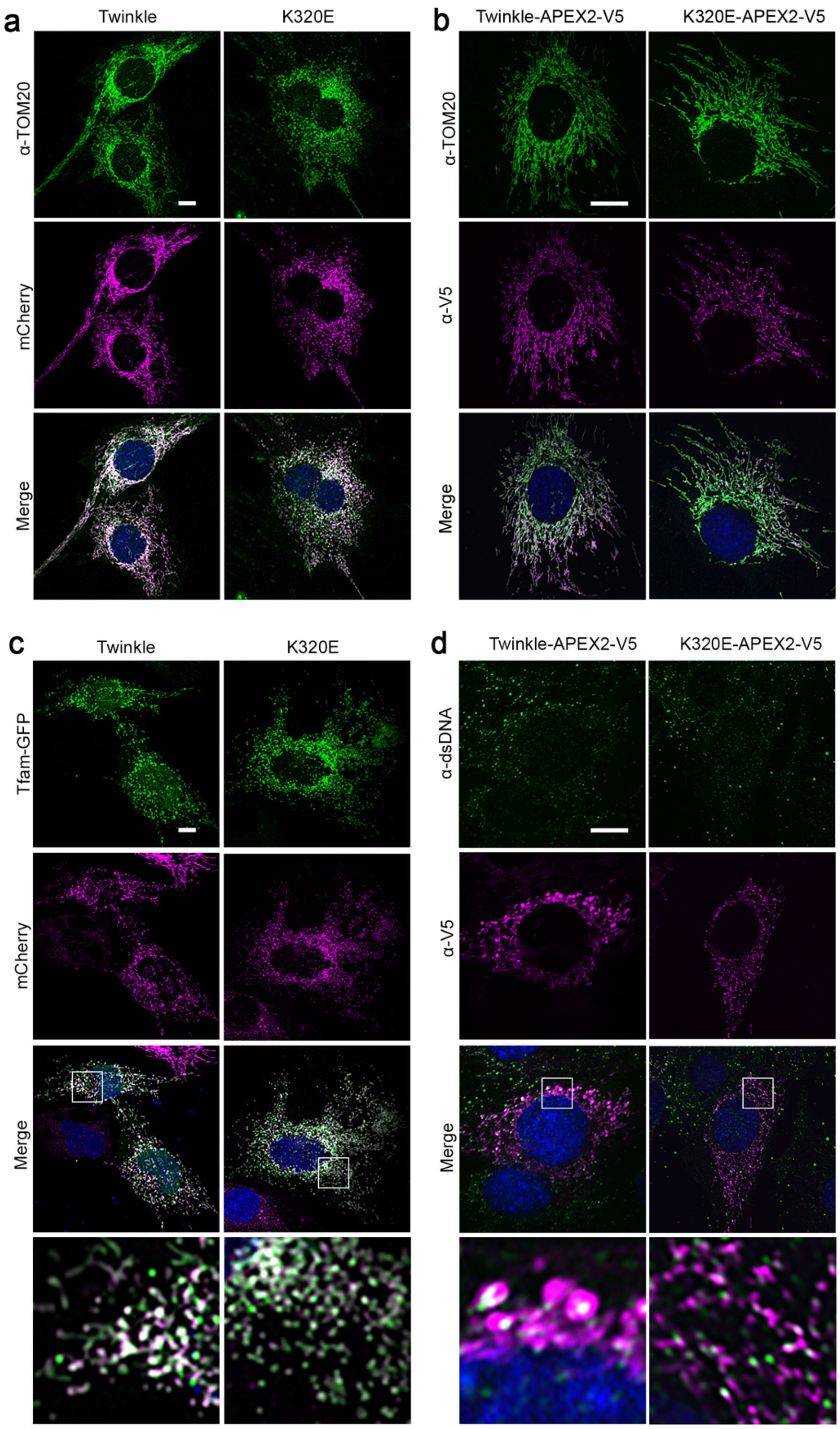

Supplementary Figure 3. (**a, b**) α-TOM20 immunofluorescence of clones expressing (**a**) m-Cherry and (**b**) APEX2-V5 tagged Twinkle variants, respectively. (**c, d**) Immunofluorescence of (**c**) m-Cherry tagged clones transiently expressing Tfam-GFP and (**d**) APEX-V5 tagged clones probed with α-dsDNA antibody showing nucleoid localization. Scale bar, 10 μm. Twinkle clones were checked regularly to maintain the expression of the transgene, either by Cherry or V5 immunofluorescence. In (**c, d**) cells were imaged in three independent experiments with similar results.

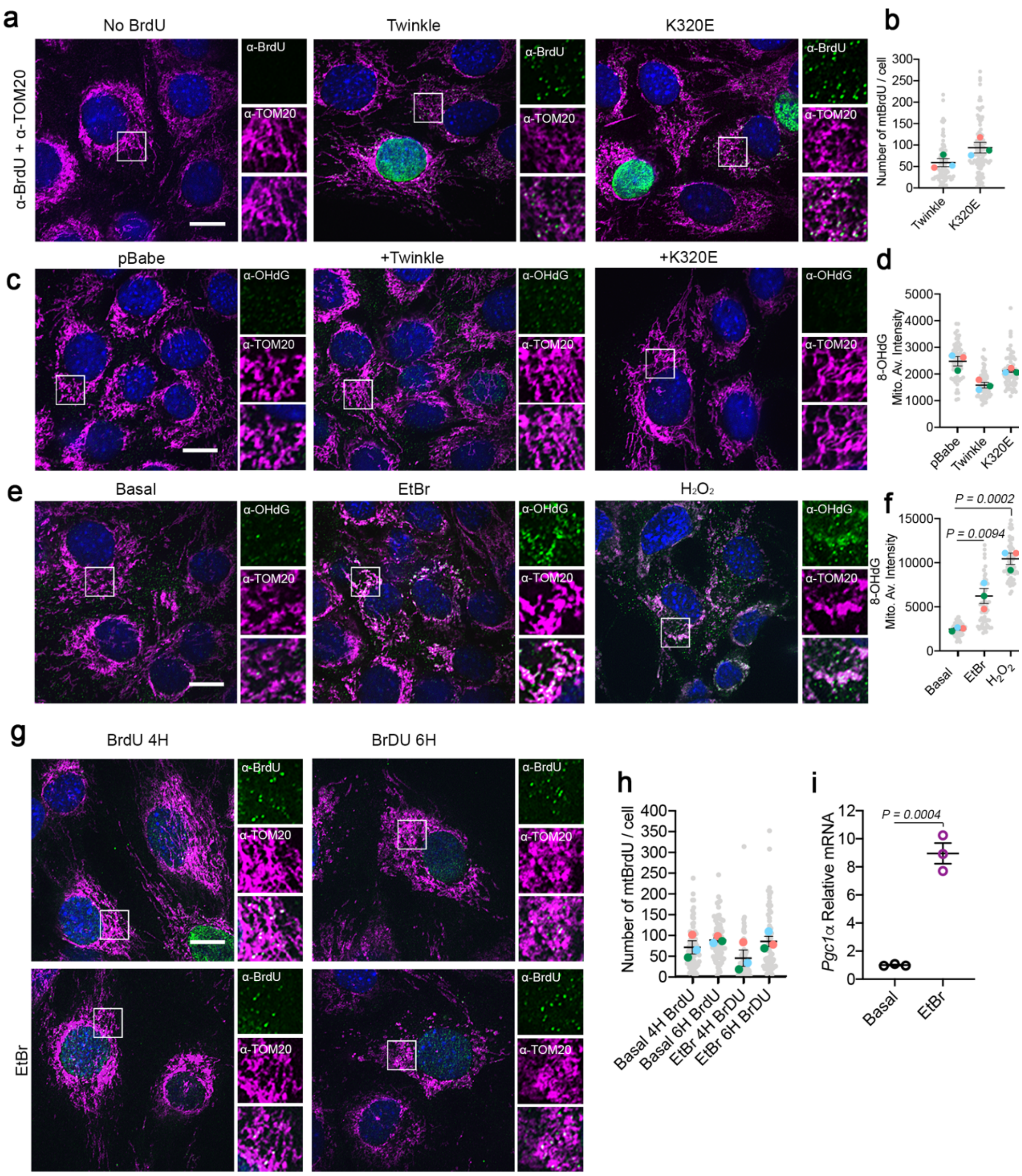

Supplementary Figure 4. (**a, b**) mtDNA replication foci analysis by α-BrdU and α-TOM20 immunofluorescence of C2C12 cells expressing Twinkle variants treated for 6h with 20 µM BrdU. Only BrdU foci inside the mitochondrial network were analysed. (n=3, >25 cells per replicate) (**c, d**) Immunofluorescence and quantification of the average intensity inside the mitochondrial network for α-OHdG and α-TOM20 binding in control C2C12 cells (n=3, >20 cells per replicate) or (**e, f**) treated 24h with 50 ng/µl EtBr or 4h with 200 µM H_2O_2 (n=3, >20 cells per replicate). (**g, h**) mtDNA replication foci analysis by α-BrdU and α-TOM20 staining of C2C12 cells incubated for 3 days with EtBr and 4 or 6h with 20 µM BrdU (n=3, >20 cells per replicate). (**i**) Quantification of *Pgc1α* mRNA in cells treated for 3 days with EtBr. *Gapdh* mRNA was used

for normalization. (n=3 independent cultures). Scale bar, 10 µm. *P* values calculated using One-way ANOVA with Tukey correction for multiple comparison (f) or Two-side unpaired Student´s T-test (i). Data is presented as Mean ± SEM.

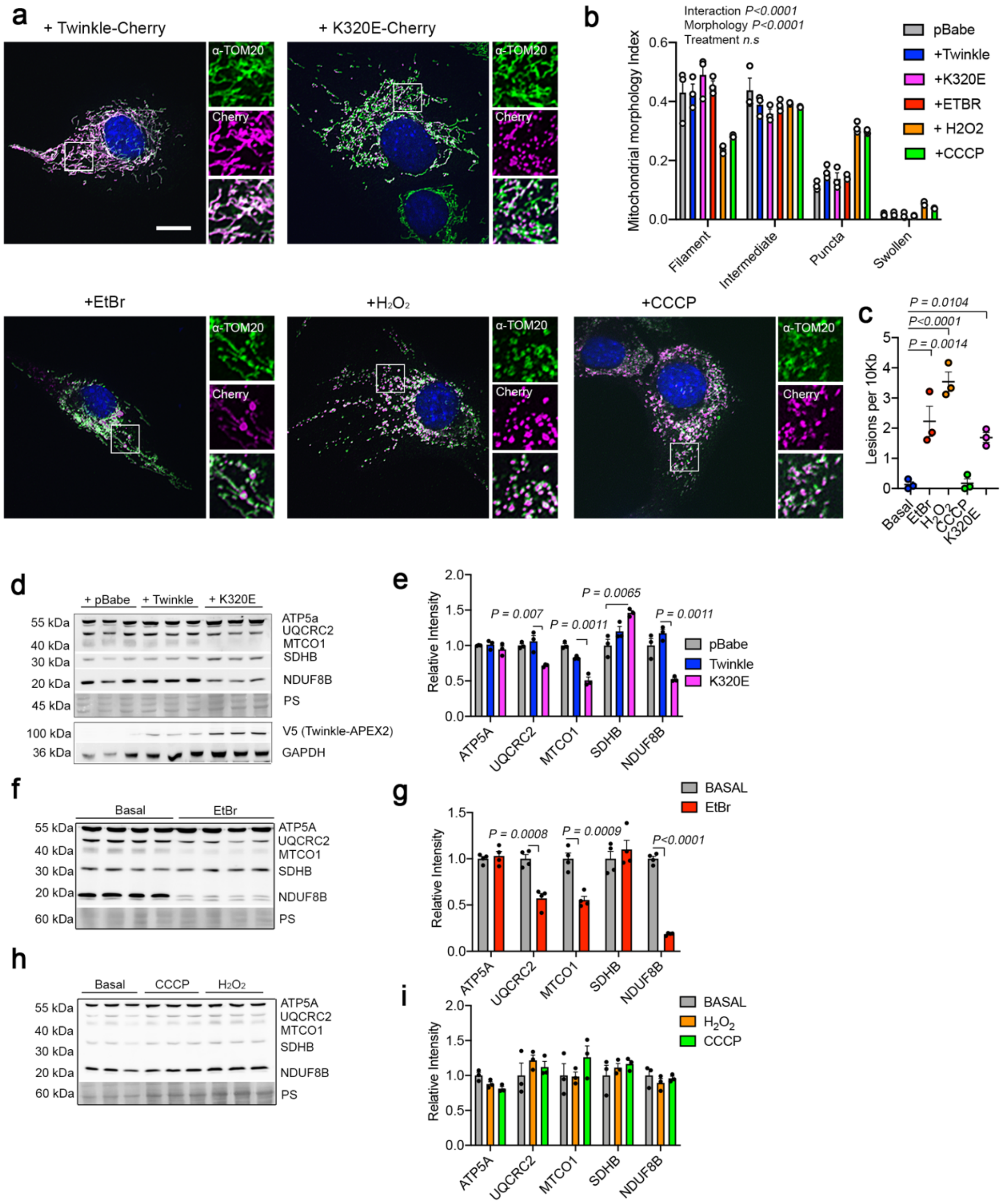

Supplementary Figure 5. (**a**) C2C12 cells expressing K320E-mCherry and Twinkle-mCherry treated with the indicated compounds and labelled with α-TOM20. Scale bar, 10 µm. (**b**) Mitochondrial morphology analysis for cells treated with the indicated mitochondrial stressors. α-TOM20 signal was used for quantification (n=3, >30 cells per replicate and condition). (**c**) Relative number of mtDNA lesions analysed by Long Run PCR in K320E cells or wt cells incubated with the indicated compounds (n=3 independent cultures). (**d-i**) Western blot and quantification of mitochondrial OXPHOS protein levels after the indicated treatments. Ponceau

S (PS) was used as a loading control and for normalization. (n=3-4 independent cultures). *P* values calculated using One-way ANOVA with Tukey correction for multiple comparison (**c**, **e** and **g**), or Two-way ANOVA for Morphology and Treatment (**b**). Data is presented as Mean ± SEM.

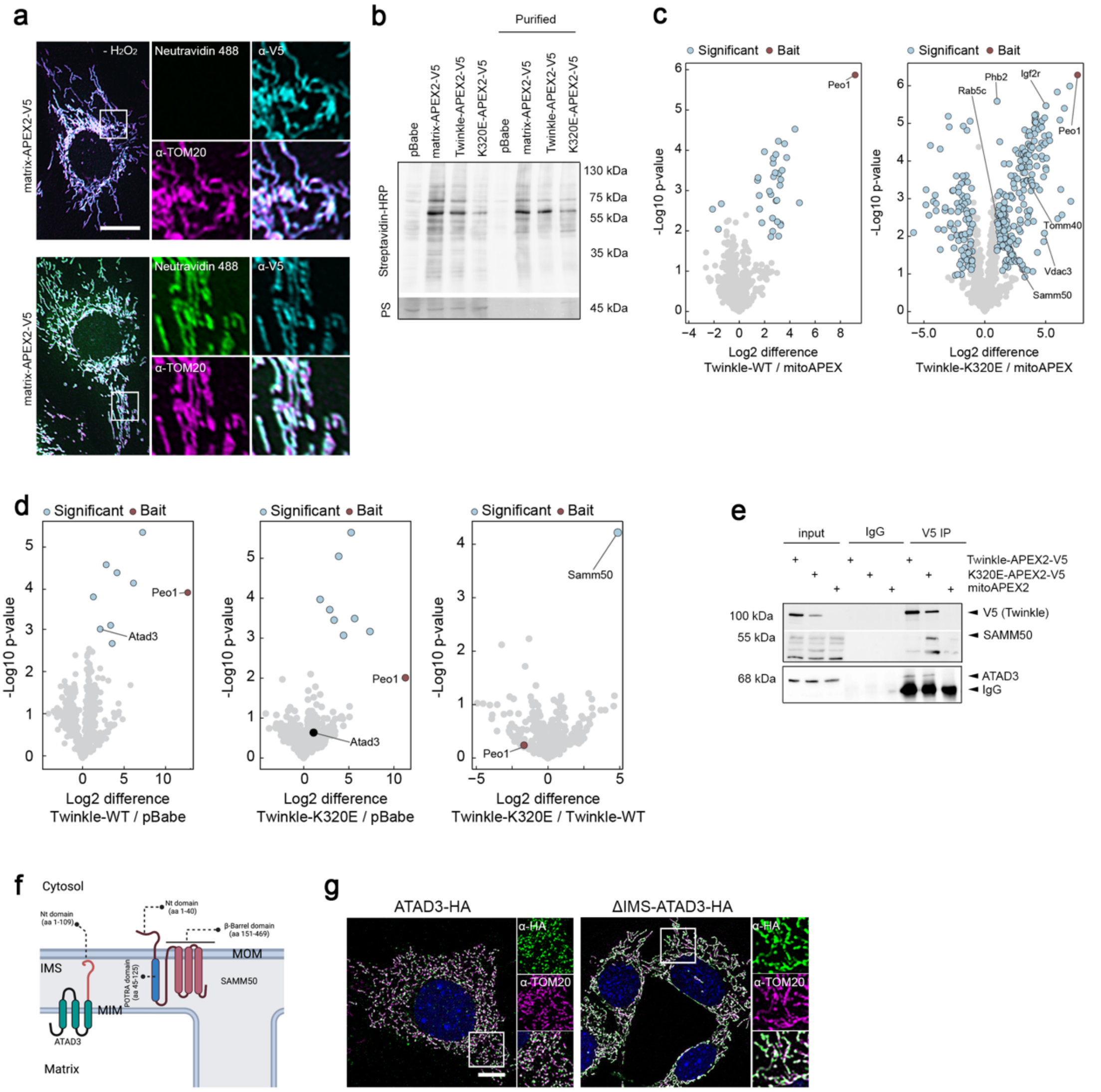

Supplementary Figure 6. (**a**) C2C12 cells expressing mito-APEX2-V5 variants exposed to biotin-phenol crosslinking. Biotinylated proteins were detected with α-Neutravidin, mitochondria with α-TOM20 and α-V5 antibodies for tagged Twinkle variants. Similar results were obtained with 4 clones obtained from independent viral transduction. (**b**) Western blot analysis of biotinylated proteins and purification of different protein extracts from the indicated cell lines. Ponceau S (PS) was used as a loading control and an α-Streptavidin-HRP antibody to detect biotinylated proteins. (**c**) Volcano plots showing proteins enriched after crosslinking and purification of Twinkle and K320E-APEX2-V5. Differentially expressed proteins compared with cells transfected with mitoAPEX2 in the matrix (significant: q-value < -0.05 and absolute log2 fold change > 1) are highlighted in blue. Twinkle (*Peo1*) is highlighted in red. (**d**) Volcano plot

showing proteins enriched after direct immunoprecipitation and MS analysis of Twinkle-APEX2-V5 and K320E-APEX2-V5 (significant: q-value < -0.05 and absolute log2 fold change > 1). (**e**) V5 co-Immunoprecipitation in steady state of Twinkle variants with ATAD3 and SAMM50. (**f**) Schematic representation of ATAD3 and SAMM50 in the mitochondrial membrane. Graph was created with a full licensed Biorender.com. (**g**) MEFs transduced with ATAD3-HA or ΔIMS-ATAD3-HA and labeled with α-TOM20 and α-HA antibodies. Images were obtained from 2 independent cultures as a control prior to biochemical experiments. Scale bar, 10 μm.

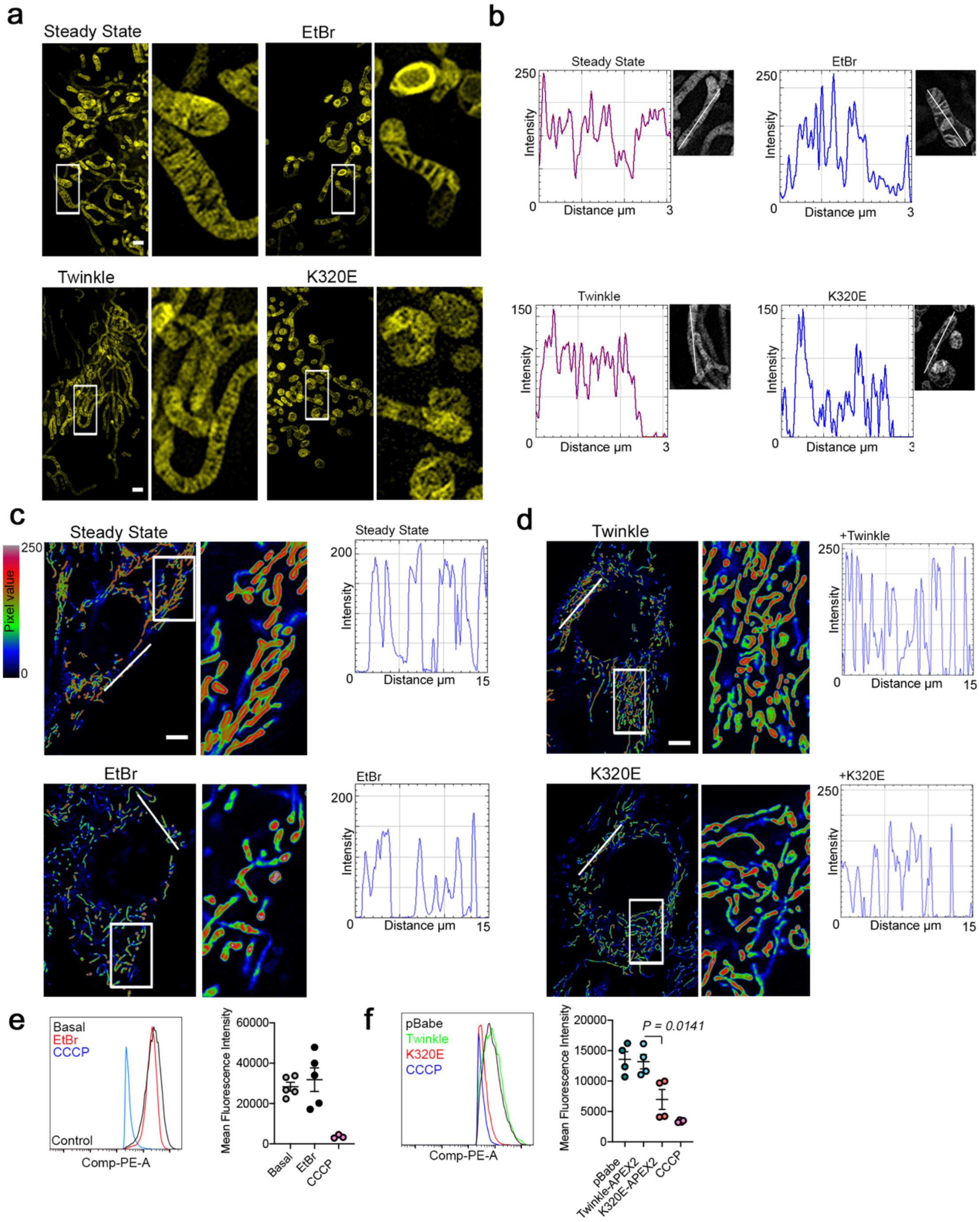

Supplementary Figure 7. (**a**) Representative images of Super-resolution gSTED microscopy for mitochondrial cristae obtained with PK Mito Orange staining. (**b**) Fluorescence profile of a cross sectioned mitochondrion (3 µm) showing differential distribution of cristae upon EtBr treatment or K320E expression. (**c**) Airy Scan Super resolution images from TMRE stained cells in steady state, treated with EtBr or (**d**) expressing Twinkle or K320E. Fluorescence profiles were obtained with 15 µm line of the indicated areas. (**e, f**) Flow cytometric analysis of mitochondrial membrane potential using TMRE (n=4 independent cultures; n=3 for CCCP analysis). Life Super-resolution microscopy for TMRE and PK Mito Orange was performed in two different days in two independent cultures per experiment with similar results. Scale bar, 1 µm (**a**) or 5 µm (**c**). *P* value obtained using One-way ANOVA with Tukey correction for multiple comparison. Data is presented as Mean ± SEM.

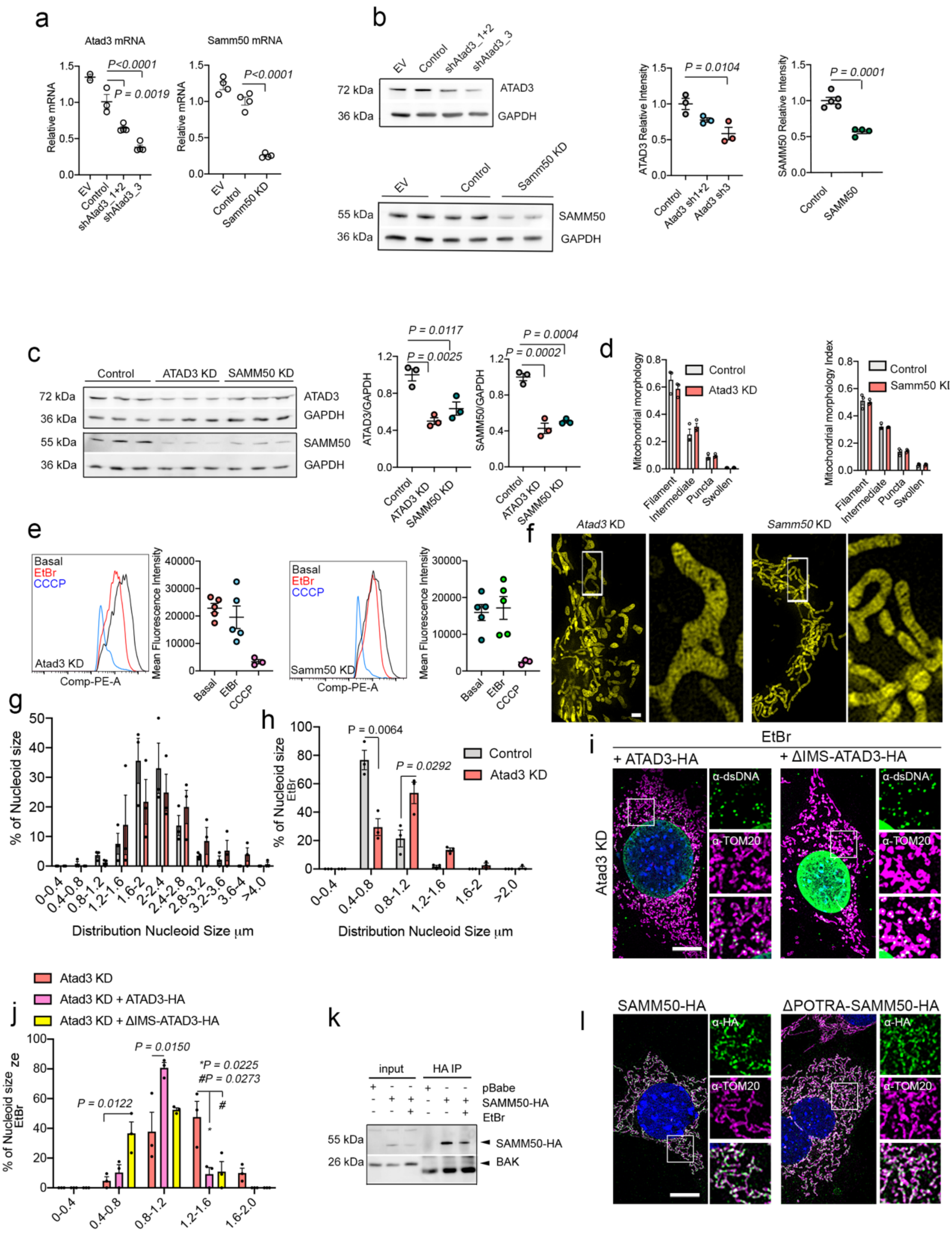

Supplementary Figure 8. (**a**) qPCR mRNA quantification of *Atad3* and *Samm50* after transduction with specific shRNAs. *Gapdh* mRNA was used for normalization (n=3 independent cultures, EV, empty vector). (**b, c**) Western blot analysis and quantification of ATAD3 and SAMM50 steady state levels upon downregulation (n=3 independent cultures). (**d**) Mitochondrial morphology analysis in *Atad3* and *Samm50 KD* cells (n=3, >40 cells per experiment). (**e**) Flow cytometric analysis of mitochondrial inner membrane potential using TMRE (n=5 independent cultures, n=3 for CCCP analysis). (**f**) gSTED Super-resolution images of mitochondrial cristae morphology using PK Mito Orange. Images were acquired in two

different days from two independent cultures per experiment with similar results. (**g**) mtDNA foci size distribution in steady state and (**h**) after EtBr treatment (n=3, >40 cells per replicate). (**i**) Immunofluorescence of α-dsDNA foci and α-TOM20 in *Atad3* KD cells transduced with ATAD3-HA or ΔIMS-ATAD3-HA and treated with EtBr. (**j**) mtDNA foci size distribution in *Atad3* KD cells transduced with ATAD3-HA or ΔIMS-ATAD3-HA. (n=3, >20 cells per replicate). (**k**) Co-immunoprecipitation of SAMM50-HA with BAK. (**l**) α-TOM20 and α-HA immunofluorescence of cells transduced with SAMM50-HA or ΔPOTRA-SAMM50-HA. Images were obtained from 2 independent cultures as a control prior to biochemical experiments. Scale bar 1 µm (**f**) or 10 µm (**i, l**). *P* value obtained using One-way ANOVA with Tukey correction for multiple comparison (**a, b, c** and **j**) or Two-side unpaired Student´s T-test (**h**). Data is presented as Mean ± SEM.

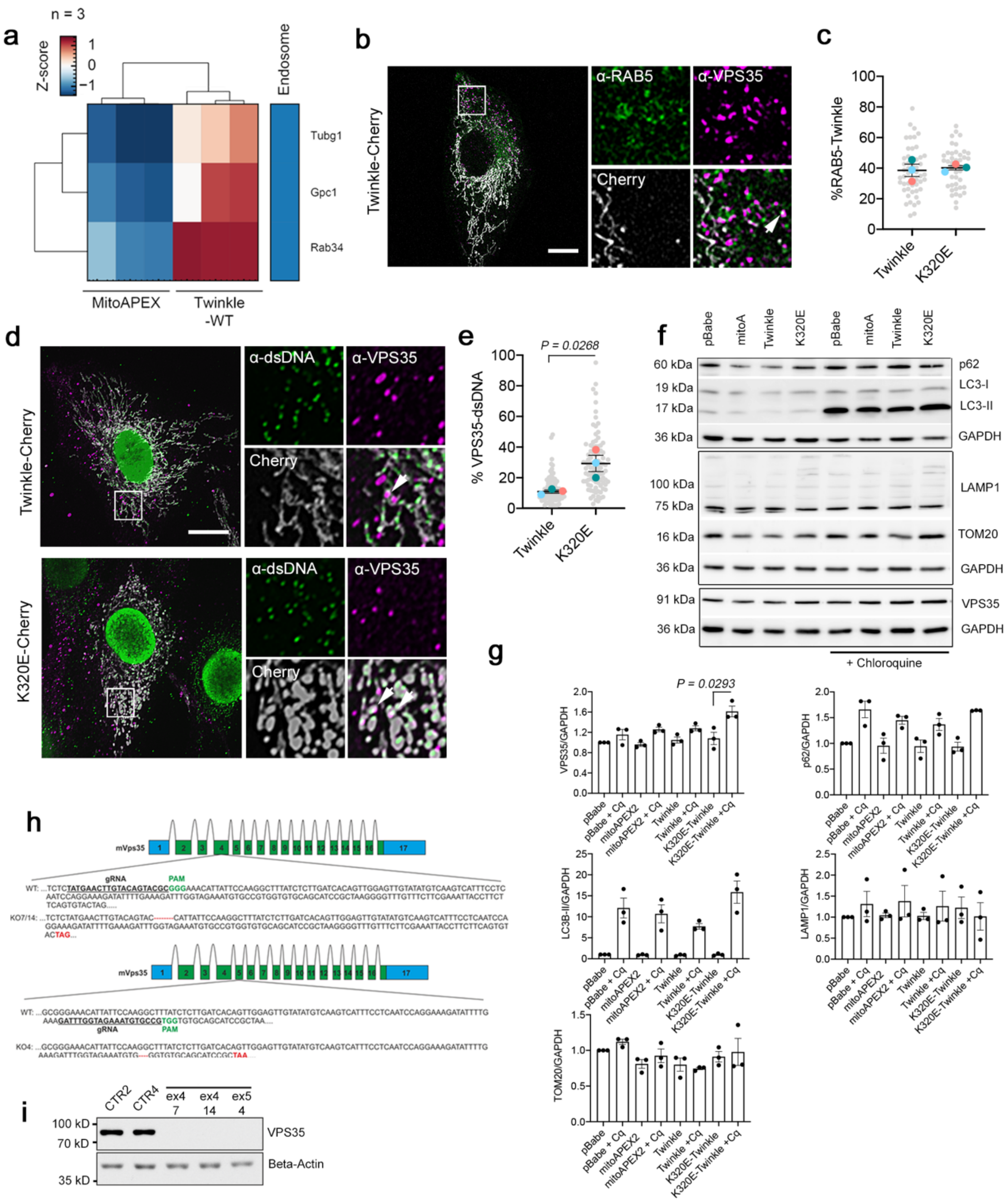

Supplementary Figure 9. (**a**) Hierarchical clustering of Z-score-normalized protein targets enrichment of proteins related to vesicle trafficking, endosomes and lysosomes for Twinkle. Targets were selected based on significant enrichments against MitoAPEX2 (significance: q-value < 0.05 and absolute log2 difference > 1). (**b-e**) Immunofluorescence and quantification of Twinkle-mCherry cells labelled with α-VPS35 and α-RAB5 or (**d**) with α-VPS35 and α-dsDNA antibodies. (n=3, >14 cells per replicate). Arrows indicate colocalization points. (**f**) Western blot analysis of autophagy markers and VPS35 levels in C2C12 cells expressing APEX2-V5 clones, treated 24h with 10 μM Chloroquine (n=3 independent cultures). (**g**) Relative intensity quantification of the indicated proteins. Values were normalized to empty vector (pBabe). (**h**) Schematic representation of DNA modification in *Vps35* Exon 4 and Exon 5, triggered by CRISPR-Cas9. (**i**) Western blot analysis of MEFs after CRISPR Cas9 mediated KO of VPS35.

Western blot analysis in *Vps35* KO cells was performed regularly to confirm the absence of VPS35 protein. Scale bar, 10 µm. *P* value obtained using Two-side unpaired Student´s T-test (**e**) or One-way ANOVA with Tukey correction for multiple comparison (**g**). Data is presented as Mean ± SEM.

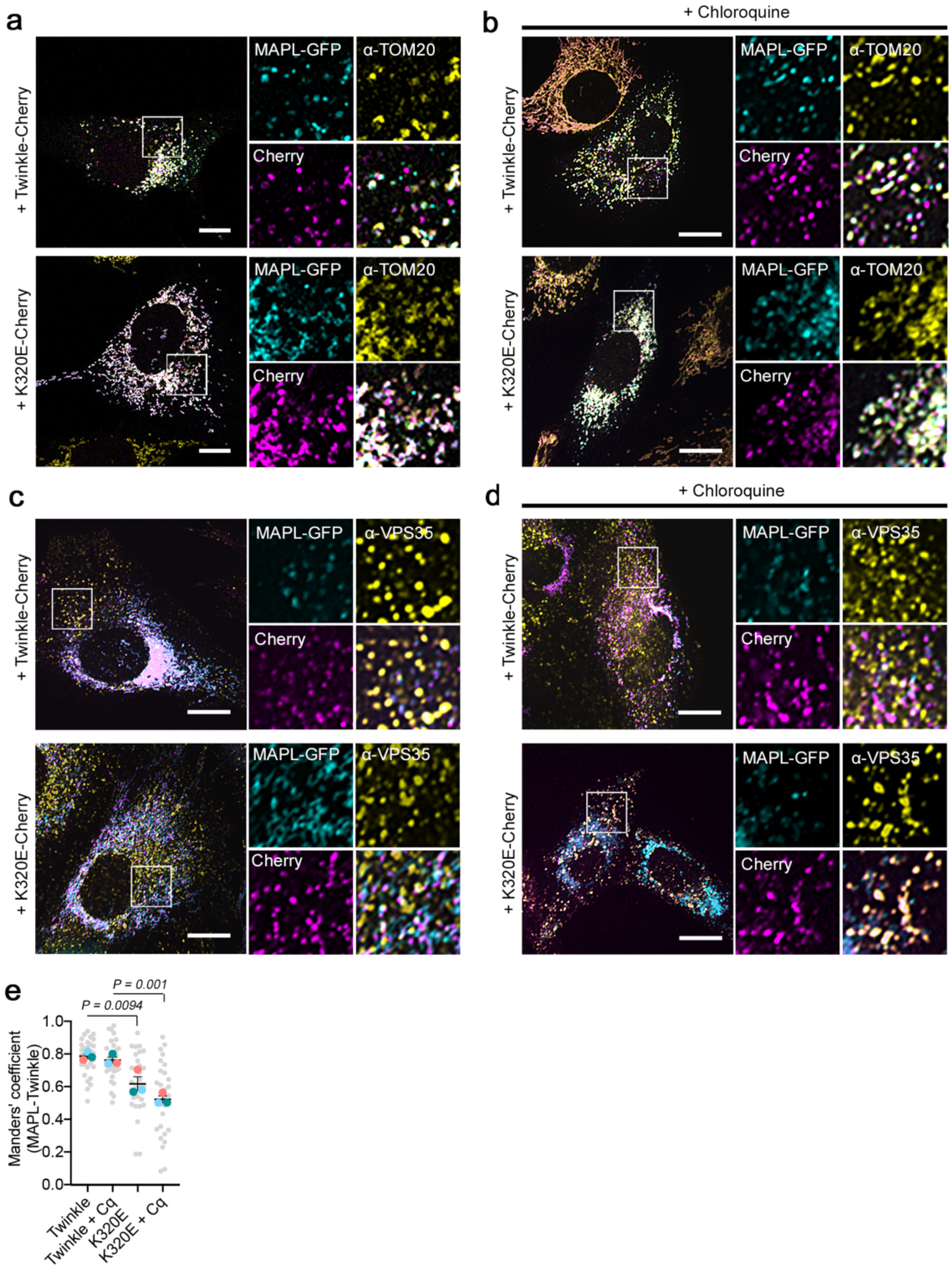

Supplementary Figure 10. (**a, b**) C2C12 cells expressing Twinkle-mCherry transiently transfected with MAPL-GFP and stained with α-TOM20 (**c**, **d**) or α-VPS35 antibody. In (**b, d**) cells were treated 4h with 10 µM Chloroquine. (**e**) Manders´ coefficient for MAPL-Twinkle

quantification (n=3, 10-15 transfected cells per replicate). Scale bar, 10 µm. *P* value obtained using One-way ANOVA. Data is presented as Mean ± SEM.

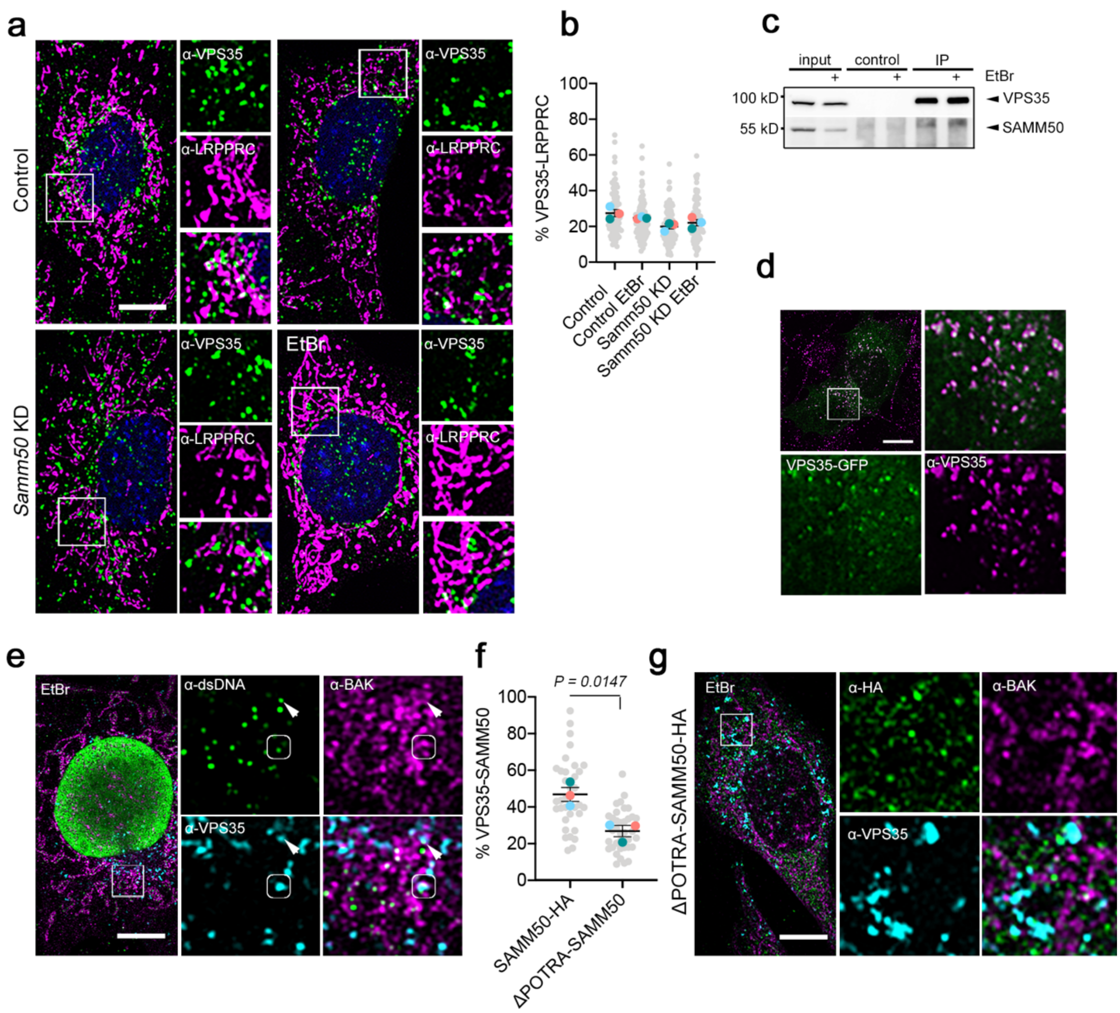

Supplementary Figure 11. (**a**) Immunofluorescence of control and *Samm50* KD cells expressing Twinkle-mCherry variants and labelled with α-LRPPRC. (**b**) Contact sites quantification of VPS35 with LRPPRC. (n=3, >30 cells per replicate). (**c**) Co-immunoprecipitation of VPS35 and SAMM50 in steady state or in EtBr treated cells. (**d**) α-VPS35 immunostaining in cells transiently transfected with VPS35-GFP and probed with α-VPS35 antibody. Images were acquired from 2 independent transfections to confirm location of the transfected protein with endogenous VPS35. (**e**) Representative Super-resolution images of EtBr treated cells labelled with α-dsDNA, α-BAK and α-VPS35. Arrows and small frames mark colocalization of three proteins (marked in three channels). Images were acquired from 3 independent cultures. (**f**) Contact sites quantification of VPS35 with SAMM50. (n=3, >12 cells per replicate in Super-resolution images). (**g**) Representative image of Airy Scan Super Resolution microscopy of cells transduced with ΔPOTRA-SAMM50-HA and labelled with α-HA, α-VPS35 and α-BAK, upon EtBr treatment. Scale bar, 10 µm. *P* value obtained using Two-side unpaired Student´s T-test. Data is presented as Mean ± SEM

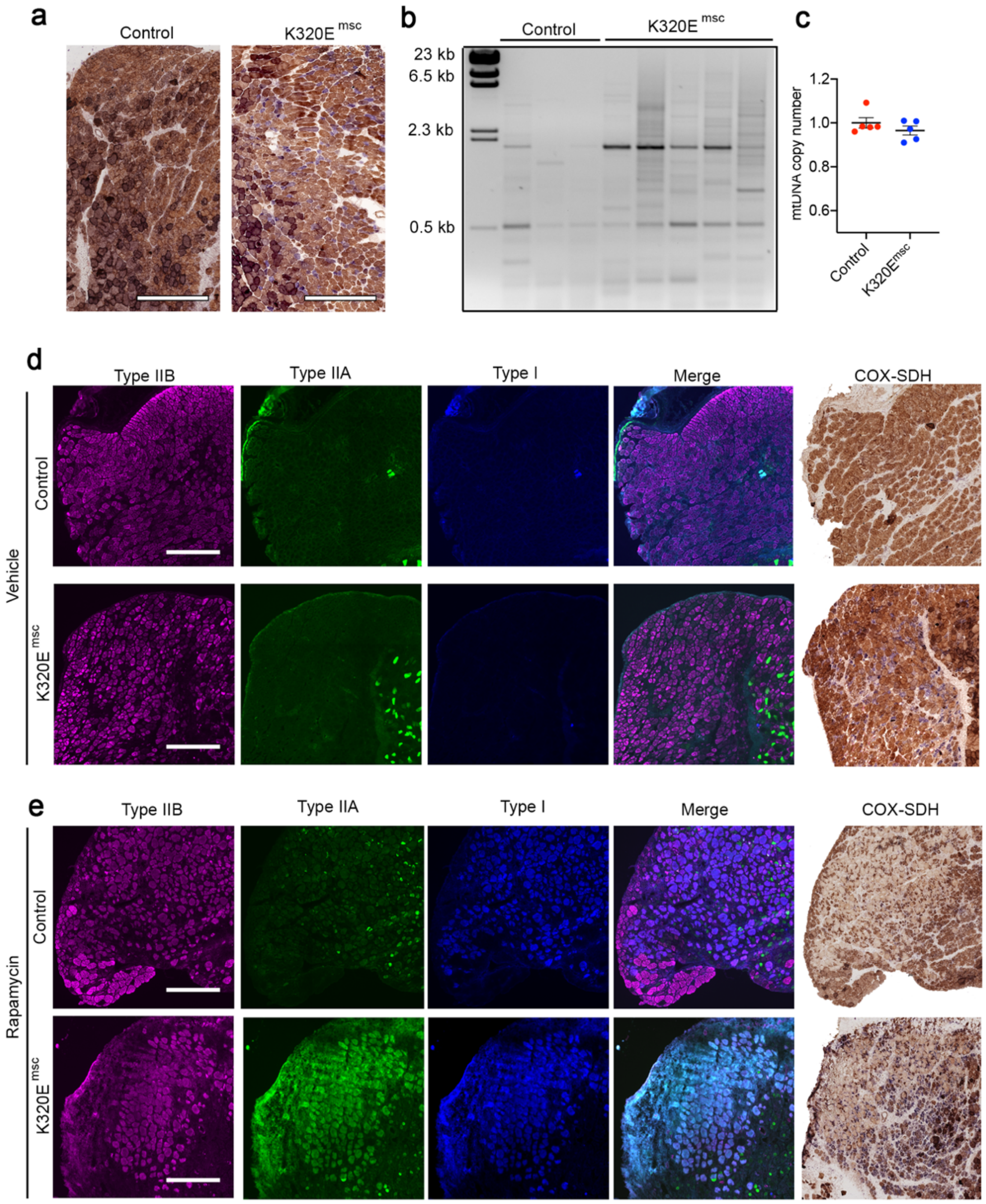

Supplementary Figure 12. (**a**) Representative image for COX-SDH activity staining in a regenerated area from the M. Tibialis anterior from control and Twinkle-K320E mice. (**b**) Long Range PCR for mtDNA deletions and, (**c**) mtDNA copy number analysis control and Twinkle-K320E mice. (n=5). Data is presented as Mean ± SEM. Muscle regeneration was induced with intramuscular injection of 10 µM Cardiotoxin (*Naja Pallida*). (**d**) Fiber type and COX-SDH serial analysis of regenerated muscles in vehicle and (**e**) rapamycin treated mice. (Control vehicle, n=4; Control rapamycin, n=3; K320E vehicle, n=6; K320E rapamycin, n=5). Scale bar, 500 µm (**a**) or 250 µm (**d** and **e**).
