# Supplementary material for: Mitochondrial membrane proteins and VPS35 orchestrate selective removal of mtDNA
Source: Nat Commun. 2022 Nov 7;13:6704. doi: 10.1038/s41467-022-34205-9 (PMC9640553; doi:10.1038/s41467-022-34205-9)
Supplement: Supplementary file 3 — Description of Additional Supplementary Files [file 41467_2022_34205_MOESM3_ESM.pdf]

File name: Supplementary Data 1

Description: Pulsed SILAC labelling in mice. Data was obtained from M. Extensor digitorum longus (EDL) and M. Soleus (SOL) from control and K320E^{msc} mice.

File name: Supplementary Data 2

Description: APEX2 proximity proteomics for Twinkle and K320E-Twinkle. Comparisons were made either with mitochondria targeted APEX2 (mitoAPEX2) or empty vector pBabe.

File name: Supplementary Data 3

Description: Dataset for Twinkle and K320E-Twinkle immunoprecipitation (IP) followed by Mass spectrometry analysis (MS).
