# Supplementary material for: Mitochondrial membrane proteins and VPS35 orchestrate selective removal of mtDNA
Source: Nat Commun. 2022 Nov 7;13:6704. doi: 10.1038/s41467-022-34205-9 (PMC9640553; doi:10.1038/s41467-022-34205-9)
Supplement: Supplementary file 7 — Reporting Summary [file 41467_2022_34205_MOESM7_ESM.pdf]

nature portfolio

Corresponding author(s): David Pla-Martín

Last updated by author(s): 13.10.2022

Reporting Summary

Nature Portfolio wishes to improve the reproducibility of the work that we publish. This form provides structure for consistency and transparency in reporting. For further information on Nature Portfolio policies, see our Editorial Policies and the Editorial Policy Checklist.

Statistics

For all statistical analyses, confirm that the following items are present in the figure legend, table legend, main text, or Methods section.

n/a	Confirmed	
☐	☒	The exact sample size (*n*) for each experimental group/condition, given as a discrete number and unit of measurement
☐	☒	A statement on whether measurements were taken from distinct samples or whether the same sample was measured repeatedly
☐	☒	The statistical test(s) used AND whether they are one- or two-sided<br>*Only common tests should be described solely by name; describe more complex techniques in the Methods section.*
☒	☐	A description of all covariates tested
☒	☐	A description of any assumptions or corrections, such as tests of normality and adjustment for multiple comparisons
☐	☒	A full description of the statistical parameters including central tendency (e.g. means) or other basic estimates (e.g. regression coefficient) AND variation (e.g. standard deviation) or associated estimates of uncertainty (e.g. confidence intervals)
☐	☒	For null hypothesis testing, the test statistic (e.g. *F*, *t*, *r*) with confidence intervals, effect sizes, degrees of freedom and *P* value noted<br>*Give P values as exact values whenever suitable.*
☒	☐	For Bayesian analysis, information on the choice of priors and Markov chain Monte Carlo settings
☒	☐	For hierarchical and complex designs, identification of the appropriate level for tests and full reporting of outcomes
☒	☐	Estimates of effect sizes (e.g. Cohen's *d*, Pearson's *r*), indicating how they were calculated

Our web collection on statistics for biologists contains articles on many of the points above.

Software and code

Policy information about availability of computer code

Data collection	No software was used for data collection
Data analysis	Western blots and microscopy images were analyzed using Image J/Fiji (https://imagej.net/software/fiji/downloads)<br>Proteomics data were analyzed and visualized with Perseus (https://maxquant.net/perseus/) and InstantClue (http://www.instantclue.uni-koeln.de)<br>Graphs and statistic analysis were performed using Graph Pad Prism.<br>Images were prepared using Adobe Photoshop Elements

For manuscripts utilizing custom algorithms or software that are central to the research but not yet described in published literature, software must be made available to editors and reviewers. We strongly encourage code deposition in a community repository (e.g. GitHub). See the Nature Portfolio guidelines for submitting code & software for further information.

Data

Policy information about availability of data

All manuscripts must include a data availability statement. This statement should provide the following information, where applicable:

- Accession codes, unique identifiers, or web links for publicly available datasets
- A description of any restrictions on data availability
- For clinical datasets or third party data, please ensure that the statement adheres to our policy

Source data for figures are provided with the paper. The data regarding proteomics analysis have been deposited to the ProteomeXchange Consortium via the PRIDE partner repository with the dataset identifier PXD023939.

Field-specific reporting

Please select the one below that is the best fit for your research. If you are not sure, read the appropriate sections before making your selection.

☒ Life sciences ☐ Behavioural & social sciences ☐ Ecological, evolutionary & environmental sciences

For a reference copy of the document with all sections, see nature.com/documents/nr-reporting-summary-flat.pdf

Life sciences study design

All studies must disclose on these points even when the disclosure is negative.

Sample size	The sample size for animal experimentation was determined empirically using G power software.<br>The sample size for each experimental condition was obtained from at least 3 different measurements in different days. In experiments involving cell image analysis, the number of cells analyzed per replicate was determined to assure representation of the whole cell population.<br>For proximity proteomics, the analysis was performed using 3 independent cultures and confirmed at least a second time with a newly generated cell line.
Data exclusions	We did not exclude samples or animals from the study.
Replication	Microscopy measurements were performed on several cell lines at different days. Error bars reflect variation within this ensemble. In regards of histology analysis using mice, several mice were used per experiment. For each mouse, at least 6 sections were stained, analyzed and averaged to obtain the value for this specific mouse.<br>For western blot analysis, experiments were carried out at different days with new set of samples every time.<br>Analysis of mtDNA was performed from samples isolated from independent cultures.
Randomization	Animals were allocated randomly to treatments. Mice from different litters were used in all experiments. Randomization was applied also for cell analysis
Blinding	For microscopy images anaylsis, the investigators were blinded to allocation and outcome assessment

Reporting for specific materials, systems and methods

We require information from authors about some types of materials, experimental systems and methods used in many studies. Here, indicate whether each material, system or method listed is relevant to your study. If you are not sure if a list item applies to your research, read the appropriate section before selecting a response.

Materials & experimental systems

n/a	Involved in the study	
☐	☒	Antibodies
☐	☒	Eukaryotic cell lines
☒	☐	Palaeontology and archaeology
☐	☒	Animals and other organisms
☒	☐	Human research participants
☒	☐	Clinical data
☒	☐	Dual use research of concern

Methods

n/a	Involved in the study	
☒	☐	ChIP-seq
☒	☐	Flow cytometry
☒	☐	MRI-based neuroimaging

Antibodies

Antibodies used	P62. Proteintech 18420-1-AP. WB 1:1000; IF 1:200<br>LC3. Proteintech. 14600-1-AP. WB 1:1000; IF 1:200<br>GAPDH. Merck. AB2302. WB 1:2000<br>OPTINEURIN. Proteintech. 10837-1-AP. WB 1:1000<br>TOM20. Proteintech. 11802-1-AP. WB 1:4000. IF 1:1000<br>V5. Thermo Scientific. R960-25. WB 1:2000; IP 1ul:100ug; IF:1:500<br>HA (mouse). Sigma. B9183. WB 1:1000; IF 1:500<br>HA (rabbit). Proteintech. 51064-2-AP. WB 1:1000; IF 1:500<br>ATAD3. Proteintech. 16610-1-AP. WB 1:1000<br>SAMM50. SCBT. sc100493. WB 1:200<br>MIC90. Proteintech. 10179-1-AP. WB 1:1000<br>OXPHOS cocktail. Abcam. ab110413. WB 1:2000<br>LRPPRC. Proteintech. 21175-1-AP. WB 1:1000<br>MIC19. Proteintech. 25625-1-AP. WB 1:1000

TOM40. Proteintech. 18409-1AP. WB 1:1000
MTCO1. Abcam. ab14705. WB 1:4000
V5. Proteintech. 14440-1-AP. WB 1:1000. IF 1:500
Streptavidin HRP. Merck. 18-152. WB 1:2000
Lamp1. Proteintech. 21887-1-AP. WB 1:500
TOM20. SCBT. Discontinued. WB 1:1000; IF: 1:500
VPS35. SCBT. sc-374372. WB 1:1000. IF 1:500; IG 1:50
SAMM50. Abcam. EPR8718. WB 1:1000
dsDNA. Abcam. ab215896. IF 1:1000
VPS35. Abcam. ab10099. IF 1:500; IG 1:200
RAB5. Abcam. ab218624. IF 1:500
BAK. Proteintech. 14673-1-AP. IF 1:200
BAK. Cell Signaling. 3814. WB 1:1000
BrdU, BD Bioscience, 550891. IF 1:1000
8-OHdG. SCBT. sc-393871. IF 1:250

MyHC-I . Developmental Studies Hybridoma Bank. BA-D5. IF: 1:100
MyHC-2A. Developmental Studies Hybridoma Bank. SC-71. IF: 1:100
MyHC-2B Developmental Studies Hybridoma Bank. BF-F3. IF: 1:100

Validation

P62. https://www.ptglab.com/products/SQSTM1-Antibody-18420-1-AP.htm
LC3. https://www.ptglab.com/products/MAP1LC3B-Antibody-14600-1-AP.htm
GAPDH. https://www.merckmillipore.com/DE/de/product/Anti-GAPDH-Antibody,MM_NF-AB2302
OPTINEURIN. https://www.ptglab.com/products/OPTN-Antibody-10837-1-AP.htm
TOM20. https://www.ptglab.com/products/TOM20-Antibody-11802-1-AP.htm
V5. https://www.thermofisher.com/antibody/product/V5-Tag-Antibody-Monoclonal/R960-25
ATAD3. https://www.ptglab.com/products/ATAD3B-Antibody-16610-1-AP.htm
SAMM50. https://www.scbt.com/p/sam50-antibody-sq-7?requestFrom=search
MIC90. https://www.ptglab.com/products/IMMT-Antibody-10179-1-AP.htm
OXPHOS cocktail. https://www.abcam.com/total-oxphos-rodent-wb-antibody-cocktail-ab110413.html
LRPPRC. https://www.ptglab.com/products/LRPPRC-Antibody-21175-1-AP.htm
MIC19. https://www.ptglab.com/products/CHCHD3-Antibody-25625-1-AP.htm TOM40. https://www.ptglab.com/products/TOMM40-Antibody-18409-1-AP.htm
MTCO1. https://www.abcam.com/mtco1-antibody-1d6e1a8-ab14705.htmlV5. https://www.ptglab.com/products/V5-tag-Antibody-14440-1-AP.htm
HA. https://www.sigmaaldrich.com/DE/de/product/sigma/h9658
HA. https://www.ptglab.com/products/HA-tag-Antibody-51064-2-AP.htm
Streptavidin HRP. https://www.merckmillipore.com/DE/de/product/Streptavidin-HRP-conjugate,MM_NF-18-152
LAMP1. https://www.ptglab.com/products/CD107a-Antibody-21997-1-AP.htm
TOM20. SCBT. Discontinued
VPS35. https://www.scbt.com/p/vps35-antibody-b-5?requestFrom=search
SAMM50. https://www.abcam.com/samm50sam50-antibody-epr8718-ab133709.html
dsDNA. https://www.abcam.com/ds-dna-antibody-dsd958-ab215896.html
VPS35. https://www.abcam.com/vps35-antibody-ab10099.html
RAB5. https://www.abcam.com/rab5-antibody-epr21801-ab218624.html
BAK. https://www.ptglab.com/products/BAK-Antibody-29552-1-AP.htm
BAK. https://www.cellsignal.com/products/primary-antibodies/bak-antibody/3814
BrdU. https://www.bdbiosciences.com/en-us/products/reagents/functional-cell-based-reagents/bromodeoxyuridine-brdu.550891
8-OHdG. https://www.scbt.com/de/p/8-ohdg-antibody-e-8
MyHC-I. https://dshb.biology.uiowa.edu/BA-D5
MyHC-2A. https://dshb.biology.uiowa.edu/SC-71
MyHC-2B https://dshb.biology.uiowa.edu/BF-F3

Eukaryotic cell lines

Policy information about cell lines

Cell line source(s)

C2C12 and HEK line was purchased in ATCC. MEF were generated in the lab from 13.5 days mouse embryos and inmortalized by transducing with antigen T from SV40 virus.

Authentication

We did not authenticate any cell line

Mycoplasma contamination

Cell lines were routinely screened using PCR to exclude mycoplasma contamination. All experiments were performed in mycoplasma free cells.

Commonly misidentified lines
(See ICLAC register)

No commonly misidentified lines were used in this study

Animals and other organisms

Policy information about studies involving animals; ARRIVE guidelines recommended for reporting animal research

Laboratory animals

Mlc1-Cre experiments for basal autophagy were performed in 24 months old mice.
Mlc1-Cre experiments for of autophagy flux and in vivo pSILAC were performed in 12 months old mice.

	Pax7-Cre regeneration experiments were performed in 6 months mice
Wild animals	No wild animals were used in this study
Field-collected samples	No samples collected in the field were used in this study
Ethics oversight	All animal procedures were performed in accordance with European Union (EU directive 86/609/EEC), national (Tierschutzgesetz), and institutional guidelines and were approved by local authorities (Landesamt für Natur, Umwelt, und Verbraucherschutz Nordrhein-Westfalen, Germany).

Note that full information on the approval of the study protocol must also be provided in the manuscript.
